# Precise Populations’ Description in Dairy Ecosystems Using Digital Droplet PCR: The Case of *L. lactis* Group in Starters

**DOI:** 10.3389/fmicb.2020.01906

**Published:** 2020-08-06

**Authors:** Marie-Aurore Caillaud, Martine Abeilhou, Ignacio Gonzalez, Marjorie Audonnet, Frédéric Gaucheron, Muriel Cocaign-Bousquet, Hélène Tormo, Marie-Line Daveran-Mingot

**Affiliations:** ^1^TBI, Université de Toulouse, CNRS, INRAE, INSA, Toulouse, France; ^2^Université de Toulouse, Ecole d’Ingénieurs de Purpan, INPT, Toulouse, France; ^3^Centre National Interprofessionnel de l’Economie Laitière (CNIEL), Paris, France

**Keywords:** *L. lactis*, *L. cremoris*, biovar diacetylactis, starters, ddPCR

## Abstract

*Lactococcus lactis* group (composed of the *lactis* and *cremoris* subspecies, recently reassigned as two distinct species) plays a major role in dairy fermentations. Usually present in starter cultures, the two species enable efficient acidification and improve the organoleptic qualities of the final product. Biovar diacetylactis strains produce diacetyl and acetoin, aromas from the citrate metabolization. As these populations have distinct genomic and phenotypic characteristics, the proportions of each other will affect the final product. Today, there is no quantitative test able to distinguish between the two species and the biovar in dairy ecosystems. In this study, we developed a specific, reliable, and accurate strategy to quantify these populations using, species-, and diacetylactis-specific fluorescent probes in digital droplet PCR assays (ddPCR). Species were distinguished based on three single nucleotide polymorphisms in the glutamate decarboxylase *gadB* gene, and the *citD* gene involved in citrate metabolism was used to target the biovar. Used in duplex or singleplex, these probes made it possible to measure the proportion of each population. At 59°C, the probes showed target specificity and responded negatively to the non-target species usually found in dairy environments. Depending on the probe, limit of detection values in milk matrix ranged from 3.6 × 10^3^ to 1.8 × 10^4^ copies/ml. The test was applied to quantify sub-populations in the *L. lactis* group during milk fermentation with a commercial starter. The effect of temperature and pH on the balance of the different populations was pointed out. At the initial state, *lactis* and *cremoris* species represent, respectively, 75% and 28% of the total *L. lactis* group and biovar diacetylactis strains represent 21% of the *lactis* species strains. These ratios varied as a function of temperature (22°C or 35°C) and acidity (pH 4.5 or 4.3) with *cremoris* species promoted at 22°C and pH4.5 compared to at 35°C. The biovar diacetylactis strains were less sensitive to acid stress at 35°C. This methodology proved to be useful for quantifying *lactis* and *cremoris* species and biovar diacetylactis, and could complete 16S metagenomics studies for the deeply description of *L. lactis* group in complex ecosystems.

## Introduction

In dairy industries, starters are simple ecosystems that transform milk into different final products depending on the biochemical and microbial composition and the technology applied. Qualitative and quantitative descriptions of these ecosystems are thus useful to monitor milk transformation. Among the microorganisms involved in food fermentations, *Lactococcus lactis* species, the primary component of starter cultures, plays a major role. During cheese making, *Lactococcus lactis* acidifies the milk, changing its physico-chemical characteristics and preventing the growth of pathogenic and spoilage bacteria. Moreover, *Lactococcus lactis* creates appropriate biochemical conditions for ripening and contributes to the final texture and flavor of cheeses (Parente and Cogan, 2004; Smit et al., 2005).

*Lactococcus lactis* species is divided into different subspecies, among which the two subspecies *lactis* and *cremoris* are of industrial interest. Before the advent of molecular methods for strain identification, these subspecies were identified using phenotypic tests (Schleifer et al., 1985). Later, several studies based on genome-wide (Kelleher et al., 2017; Torres Manno et al., 2018) and comparative analysis of ANI parameters (Cavanagh et al., 2015a) support the idea that these two subspecies can be considered as two distinct species *lactis* and *cremoris*. The use of this new denomination is now well admitted. In the *lactis* group, both species are associated with well described growth characteristics (Wels et al., 2019). However, *cremoris* species have different phenotypes (Cavanagh et al., 2015a), with strains identified as “true” *cremoris*, and strains showing a Lactis phenotype (growth at 40°C, in the presence of 4% NaCl and pH 9.2, and the ability to degrade arginine). Dairy starters are usually a mix of strains of the two genotypes. The proportion of each genotype is important because it affects the characteristics of the final product (Fernández et al., 2011). Several methods are available for the identification of *lactis* and *cremoris* genotypes (Cavanagh et al., 2015b), including glutamate decarboxylase activity in *L. lactis* species (Nomura et al., 1999). In *L. cremoris*, the non-functional activity is related to a frameshift mutation in the glutamate decarboxylase gene, *gadB*, thus encoding a non-functional protein (Nomura et al., 2000). The study that followed these works produced a successful method to distinguish the two reassigned species, based on PCR amplification and RFLP analysis of the *gadB* gene in pure cultures (Nomura et al., 2002).

Among *lactis* species, the biovar diacetylactis is usually of considerable interest for the dairy industry as it includes strains that produce diacetyl and acetoin, thus improving product flavor by adding a creamy or buttery aroma to the final products. This aroma is related to citrate metabolism. Citrate is uptaken by a citrate permease encoded by the plasmidic *citP* gene (García-Quintáns et al., 1998). After internalization, citrate is converted into oxaloacetate and acetate by the citrate lyase, an enzyme encoded by the chromosomic *citDEF* genes. Finally, oxaloacetate is decarboxylated into pyruvate, leading to C4 compounds among which diacetyl and acetoin. The citrate-fermenting capacity of the biovar diacetylactis strains is usually identified by the growth of blue colonies on Kempler and McKay medium (Kempler and McKay, 1980) or by the presence of the PCR amplified *citP* gene, systematically associated with the presence of the chromosomal cluster (Passerini et al., 2013).

To our knowledge, no quantitative test has yet been developed to quantify *cremoris* and *lactis* species or the biovar diacetylactis population in dairy ecosystems. Specific identification and quantification require a reliable and accurate strategy. PCR-based assays using TaqMan probes technology are suitable to detect specific targets of interest (Law et al., 2015). This strategy can be very useful to distinguish nucleotide variants with single nucleotide polymorphism (SNP) precision (Billard et al., 2012; Strayer et al., 2016). However, the choice of the target is a crucial step to ensure the reliability, and the accuracy of the detection and the quantification.

Quantitative PCR assays (qPCR or real-time PCR) are widely used to describe microbial communities in the environment (Smith and Osborn, 2009) or in food samples (Tessonnière et al., 2009; Zago et al., 2009; Postollec et al., 2011). However, qPCR has some limitations for the absolute quantification (Morisset et al., 2013; Ren et al., 2017; Gobert et al., 2018). The recent digital droplet PCR (ddPCR) technology has been particularly successful in clinical diagnosis or in detecting foodborne pathogens (Elizaquível et al., 2012; Day et al., 2013). In fact, ddPCR is more accurate, sensitive, precise and resistant to inhibitors (Wang et al., 2018) than qPCR. Moreover, the end point measurement and the partitioning technology allow the absolute quantification in copies per microliter, based on Poisson distribution, without a need for standard curves.

The aim of this study was to develop a reliable, fast, sensitive, precise and specific method to describe and quantify the proportions of the two species and the biovar diacetylactis of *L. lactis* group in dairy ecosystems like starters. The method was used to monitor variations in different subpopulations in a commercial starter during the production of a fermented milk under different pH (4.5 and 4.3) and temperature (22°C or 35°C) conditions.

## Materials and Methods

### Bacterial Strains and Culture Conditions

All analyzed strains of *Lactococcus lactis*, *Lactococcus cremoris*, *Lactococcus lactis* biovar diacetylactis, *Streptococcus thermophilus, Lactococcus garvieae, Enterococcus faecalis, Enterococcus faecium, Leuconostoc mesenteroïdes, Leuconostoc citreum, Leuconostoc faecium* and *Leuconostoc lactis* are listed in Table 1.

**TABLE 1:** List of genus, species and strains tested to develop the test.

**Genus**	**Species**	**Biovar**	**Strain**	**References**
*Lactococcus*	*cremoris*		189	From Sandine lab
	*cremoris*		188	From Sandine lab
	*cremoris*		HER 1203	From Ackermann lab
	*cremoris*		S 72	
	*cremoris*		S 78	
	*cremoris*		S 82	
	*cremoris*		S 102	
	*cremoris*		S 103	
	*cremoris*		S 105	
	*cremoris*		HER 1205	From Ackermann lab
	*cremoris*		EIP 58E	Unpublished
	*cremoris*		CNRZ 268	From Novel lab
	*cremoris*		EIP 36F	Unpublished
	*cremoris*		EIP 53A	Unpublished
	*cremoris*		EIP 53I	Unpublished
	*cremoris*		L 24	From Novel lab
	*lactis*	Diacetylactis	EIP 33F	Unpublished
	*lactis*	Diacetylactis	IL 1403	Bolotin et al. (2001)
	*lactis*	Diacetylactis	EIP 33H	Unpublished
	*lactis*	Diacetylactis	UCMA 5716	Passerini et al. (2010)
	*lactis*		NCDO 2727	Obis et al. (2001)
	*lactis*		S 188	Passerini et al. (2010)
	*lactis*		EIP 13D	Unpublished
	*lactis*		CIRM-BIA2008	Obis et al. (2001)
	*lactis*		EIP 55H	Unpublished
	*lactis*		EIP 19S	Unpublished
	*lactis*		EIP19F	Unpublished
*Enterococcus*	*faecalis*		CIP 104055	
	*faecalis*		CIP 104056	
	*faecium*		CIRM-BIA499	
*Leuconostoc*	*citreum*		CIRM-BIA852	
	*citreum*		CIRM-BIA1453	
	*lactis*		CIRM-BIA1088	
	*lactis*		CIRM-BIA1450	
	*mesenteroïdes*		CIRM-BIA435	
	*mesenteroïdes*		CIRM-BIA1183	
	*faecium*		ST5A1	
*Lactococcus*	*garvieae*		DSM 20684	
	*garvieae*		DSM 29394	
*Streptococcus*	*thermophilus*		TIL868	
	*thermophilus*		TIL1359	
	*thermophilus*		TIL1421	

All the species were grown in GM17 medium at 30°C or 37°C for *Streptococcus thermophilus* strains, except for *Leuconostoc* species which were cultivated in MRS broth at 30°C.

#### Reconstituted Starters

Mixtures of the two strains were reconstituted by combining IL1403 and S72 strains (Table 1). Each strain was grown in GM17 medium overnight at 30°C. This preculture was used to inoculate a new GM17 medium at OD_580__*nm*_ = 0.05. The OD_580__*nm*_ was measured during cultivation and growth was stopped at the beginning of the stationary phase (3.85 < OD_580__*nm*_ < 4.29). Five mixtures were produced with theoretical ratios (v/v) of IL1403 to S72 of 5:95, 20:80, 50:50, 80:20 and 95:5. After centrifugation at 13,000 rpm for 5 min, the pellets were recovered and stored at −20°C until DNA extraction. The experiment was independently repeated three times.

#### Commercial Starter

MM100 commercial starter (DANISCO, CHOOZIT MM 100 LYO 25 DCU, PD 207167-10.0FR, N°50434) is composed of *Lactococcus lactis*, *Lactococcus cremoris* and *Lactococcus lactis* biovar diacetylactis. The proportion of each species, which affects the features of the fermented product, is not provided by the supplier, but the technological characteristics are specified for use at 35°C. The lyophilized starter was re-hydrated at the concentration of 25 DCU/l in peptone water and then diluted at 5 DCU/100l in 10% (w/v) sterile skimmed milk (Sigma-Aldrich) and left to grow at 22°C or 35°C until the pH reached 4.5 or 4.3. Temperature and pH ranges were closely linked to specific production processes of fermented milks and fresh soft cheeses. At each temperature, the acidification of two independent cultures was measured using the Cinac system (AMS Alliance France) (Spinnler and Corrieu, 1989) and showed highly reproducible curves that were consequently averaged. At both temperatures and at three time points during the process, before growth (T0) and after growth until pH reached 4.5 (T1) and then 4.3 (T2), the MM100 starter was characterized for *L. lactis* populations with ddPCR assays.

### DNA Extraction

DNA of all strains and reconstituted starters were extracted using the GenElute Bacterial Genomic DNA Kit (Sigma-Aldrich) according to the manufacturer’s instructions. The DNA of the fermented milks was extracted with DNeasy PowerFood Microbial Kit (Qiagen) according to the manufacturer’s instructions with the following modifications: first, the cell pellet was resuspended in the MBL solution and incubated at 70°C for 10 min. The genomic DNA concentration (C_*ng/*__μ__l_) was obtained by measuring absorbance at 260 nm on a Nanodrop ND2000. For the sake of convenience, C_*ng/*__μ__l_ was converted into C_copies/__μ__l_ according to the following equation: C_copies/__μ__l_ = Na × C_*ng/*__μ__l_ × 10^–9^/(*L* × 660), where Na is the Avogadro constant (6.02 × 10^23^/mol, and *L* is the average size of a lactococcal genome (2.5 × 10^6^ bp). This calculation provides only an estimated value in copies/μl since A260 measurement depends on various factors and does not distinguish between intact and fragmented targets.

DNA purity was evaluated by the ratio of absorbance 260 nm/280 nm. Extracted DNA was diluted and used for the ddPCR assays at appropriate dilutions.

### Primers and Probes for ddPCR

Sequences of the *gadB* gene available on the National Centre for Biotechnology Information (NCBI)^1^ were retrieved and aligned using ClustalW in MEGA7 (Kumar et al., 2016). SNPs specific to the *lactis* and *cremoris* species (positions 1332, 1335, and 1336 in IL1403 chromosome) and a conserved region in the *L. lactis* group (position 1283), were chosen to design specific hydrolysis probes. Two different fluorophores (HEX and FAM) were used to enable multiplex ddPCR. Locked nucleic acids (LNAs) were added to enhance oligonucleotide melting temperature and probe affinity and specificity (Letertre et al., 2003; Mouritzen et al., 2003). The conserved regions surrounding these probes were used to design specific primers allowing the amplification of a 149 bp fragment for the ddPCR experiments. Melting temperature predictions and the thermodynamic parameters of the primers and probes were calculated using the IDTs oligo-analyzer tools^2^. The primers, probe sequences and amplicon size are listed in Table 2. Primers were synthesized by Eurofins (Ebersberg, Germany) and probes by Eurogentec (Angers, France). All primers and probes were diluted in ultra-pure water at a concentration of 100 μM and solutions were stored at −20°C.

**TABLE 2:** Primers/probe(s) sets targeting the *gadB* and *citD* genes of *L. lactis* and *L. cremoris* species.

**Target gene**	**Target genotype**	**Primer or probe name**	**Sequence (5′–3′)**	**Amplicon or probe length (bp)**
*gadB*		Forward F-gadB	TGGAAAATGAAATCATTCAACG	149
		Reverse R-gadB	ATATGTTTTATTTTCAGGTTCCTS	
	*lactis*	P_lac_	**FAM**-TTATTTA**+A+A**GC**+C**TCA ATTGCTTCTTG-BHQ1	26
	*cremoris*	P_cre_	**FAM**-TTATTTA**+G+T**GC**+A**TCAATTGCTTCTTG-BHQ1	26
	*lactis* and *cremoris*	P_*tot*_	**HEX**-ACATAGTTAAA**+T+G+C**CATATTCATCC-BHQ1	25
*citD*		Forward F-citD	TGGTAAGCAAATCGAGGC	134
		Reverse R-citD	CACGTTCCACTACCGC	
	biovar diacetylactis	P_cit_	**FAM**-AGCTTATGCCATTGAAAATGCGGAC-BHQ1	25

*HEX, hexachlorofluorescein; FAM, 6-carboxyfluorescein; BHQ, black hole quencher; **+N** in bold, locked nucleic acid, also corresponding to SNPs positions in P_*lac*_ and P_*cre*_ FAM-probes.*

### Droplet Digital PCR (ddPCR) Assays and Procedure

ddPCR was performed with the QX200 AutoDG Droplet Digital PCR system (Bio-Rad) according to the manufacturer’s protocol. The ddPCR mastermix had a final volume of 22 μl and contained 2.2 μl of DNA, 11 μL of 2x ddPCR Supermix for Probes (No dUTP) (Bio-Rad, Ref catalog 186-3024), 1.1 μl of 18 μM forward plus reverse primers mixture, 1.1 μl of 5 μM HEX and FAM probes or 1.1 μl of 5 μM FAM probe for singleplex assay (Table 2), and 5.5 μl or 6.6 μl of nuclease free water, for the duplex or singleplex assay, respectively. Only 20 μl of the mixture was used to generate droplets using the QX200 Droplet generator. Subsequently, the droplet suspension was transferred into 96-wells plate and PCR amplification was performed on a T100 Touch thermal cycler (Bio-Rad) with the following program: 10 min at 95°C followed by 40 cycles of 30 s at 94°C and 60 s at 59°C, ending with 10 min at 98°C, at a ramp rate of 2°C/s. After PCR, each reaction was analyzed by the droplet reader (Bio-Rad) and acquired data were treated with QuantaSoft software V1.7 (Bio-Rad). Thresholds were set manually considering the double negative droplets cluster from the negative control and the FAM- and/or HEX-specific positive droplets cluster from the control strains.

The DNA concentration must be in the dynamic range of the ddPCR (0.25 copies/μl to 5,000 copies/μl) (Droplet Digital PCR Applications Guide, Bio-Rad). DNA is often diluted to obtain one theoretical copy per droplet. For limits of detection and quantification (LoD and LoQ) assays, five dilutions were tested, from the initial dilution by serial diluting to the tenth.

### Statistical Analysis

Statistical data analysis was carried out using the R statistical programming environment (R Development Core Team, 2005). The effect of pH on the *lactis* (P_lac_/P_*tot*_) and *cremoris* (P_cre_/P_*tot*_) species and the biovar diacetylactis (P_cit_/P_lac_) during milk fermentation at two different temperatures, 22°C and 35°C, was evaluated using a one-way analysis of variance (ANOVA). A further two-way ANOVA was carried out on P_lac_/P_*tot*_ and P_cre_/P_*tot*_ ratios to assess the effects of interactions between pH (4.5, 4.3) and temperature (22°C, 35°C). Normality of the samples was checked using the Shapiro-Wilk test, which showed no evidence of significant differences to a normal distribution, and homogeneity of the variances was checked with the Levene test. Conventional ANOVA with Tukey’s *post hoc* test was used when variances were homogeneous, whereas Welch’s ANOVA and the Games-Howell *post hoc* test were used when variances were heteroscedastic. Average P_lac_/P_*tot*_, P_cre_/P_*tot*_ and P_cit_/P_lac_ ratios were calculated from n independent sample measurements: *n* = 6 at the initial condition, *n* = 8 and *n* = 9 at 22°C and 35°C, respectively, and at pH 4.5, and *n* = 6 at each temperature and pH 4.3.

## Results

### Judicious Selection of Target Genes to Identify and Quantify the *L. lactis*, *L. cremoris*, and Biovar Diacetylactis Populations

**FIGURE 1:**
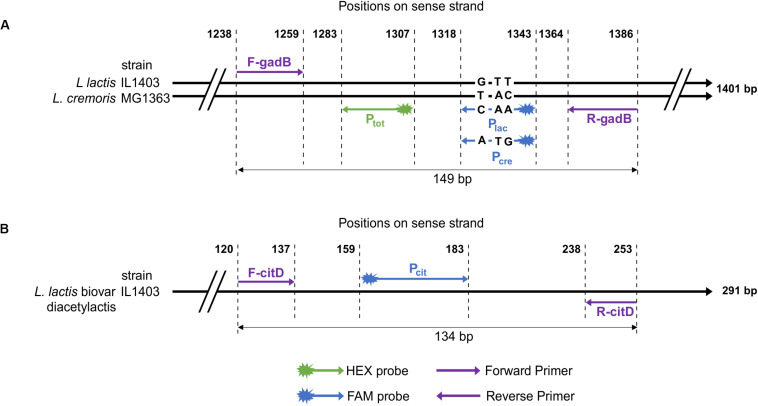
Schematic representation of the positions of primers and probes for: **(A)** *gadB* gene of two *Lactococcus* strains. IL1403 and MG1363 strains belong, respectively, to *lactis* and *cremoris* species. Positions are aligned on the 1401 bp sequence of the *gadB* gene of IL1403 strain. Nucleotides between positions 1318 and 1343 correspond to *lactis* and *cremoris* genotypes probes. **(B)** *citD* gene sequence of IL1403 strain.

The glutamate decarboxylase gene (*gadB*) was used in previous studies to distinguish the two phylogenetic groups corresponding to *lactis* and *cremoris* genotypes among the *L. lactis* strains (Nomura et al., 2002). However, the PCR-restriction fragment length polymorphism targeting *gadB* does not enable quantification of the two genotypes in simple or complex ecosystems. In order to identify specific SNPs in this gene, nucleotide sequences from 57 strains, including 21 *lactis* genotype and 36 *cremori*s genotype strains (Supplementary Table S1), were aligned. Three SNPs (positions 1332, 1335, and 1336 in IL1403 gene) were genotype-specific and served to design two probes: P_lac_ (*lactis* species) and P_cre_ (*cremoris* species). A region with no polymorphism was used for the design of primers (F-gadB and R-gadB) and a consensus probe P_*tot*_ *L. lactis* group-specific (Figure 1A). Because the droplet reader allows fluorescence reading at two wavelengths, duplex reactions were tested (FAM-P_lac_ and HEX-P_*tot*_ or FAM-P_cre_ and HEX-P_*tot*_ probes) to detect and determine the proportions of each species in the total *Lactococcus lactis* population present in a sample.

Most descriptions of the biovar diacetylactis include citrate-utilizing strains. This metabolism has been investigated in detail in the dairy strain CRL264 (Zuljan et al., 2016). The citrate is transported by the plasmid-encoded citrate permease CitP, after which its transformation into oxaloacetate and pyruvate is mediated by a chromosomal cluster encoding *citDEFXG* and *citM*, respectively (de Felipe et al., 1995). The *citD* gene encoding the citrate lyase acyl carrier protein was targeted to identify and quantify this biovar. This gene sequence is highly conserved among the biovar and regions with no polymorphism were chosen to design primers, R-citD and F-citD, and a FAM-probe, FAM-P_cit_, from 11 sequences retrieved from the GenBank database (Figure 1B and Supplementary Table S2).

### Test Development

A thermal-gradient from 65°C to 55°C was used to establish the optimal hybridization temperature. Each set of primers/probe(s) was tested on both target and non-target strains and gradient results were compared. A temperature of 59°C that specifically dissociated specific positive droplets from the negative ones was chosen for all primers/probe(s) sets.

#### Specificity of Probes

The specificity of primers and probes was tested first *in silico* using the Nucleotide Basic Local Alignment Search Tool^3^. Specificity was then further tested experimentally using DNA from samples of target and non-target species (Table 1). The selected non-target species corresponded to phylogenetically closely related species or species commonly present in the dairy environment. Firstly, the test was conducted at 59°C on 15 non-target strains belonging to *Streptococcus thermophilus, Lactococcus garvieae, Enterococcus faecalis, Enterococcus faecium, Leuconostoc mesenteroïdes, Leuconostoc citreum, Leuconostoc faecium*, and *Leuconostoc lactis* species. No cross amplification was detected in any of the three primers/probe(s) sets. Next, 27 target strains (16 *L. cremoris* and 11 *L. lactis* among which 4 biovar diacetylactis) were tested for each primers/probe(s) set. P_*tot*_, P_lac_, P_cre_, and P_cit_ only showed positive results with their target strains, thereby confirming the specificity of the probes.

#### Comparison Between Singleplex and Duplex Assays

As the test was designed to quantify the ratio of each species among the total *L. lactis* group, two probes were mixed in duplex in the reaction. The aim was to check whether the quantification of one specific target (P_lac_ or P_cre_) is affected by the presence of the consensus target (P_*tot*_) and *vice versa*. Five technical replicates were tested for *L. lactis* EIP33F strain and for *L. cremoris* HER1205 strain, with P_lac_, P_cre_, and P_*tot*_ in singleplex (a specific probe and a consensus probe in two different wells) or in duplex (a specific probe plus a consensus probe in the same well). For each probe or pair of probes, no significant variation of the measured target copy number was observed between the duplex and the singleplex assays (Table 3). The difference between the duplex and the singleplex average values was lower than the coefficient of variation representing inter-well variation in the same condition (duplex or singleplex).

**TABLE 3:** Comparison between singleplex and duplex assays.

**Probe**	**Assay**	**Target species**	**Average concentration of five replicates (copies/μ l)**	**Coefficient of variation representing inter-wells variation (%)**	**Deviation between duplex and singleplex average values as a % of the duplex value**
P_cre_	Duplex	*cremoris*	1748	5.1	0.46
	Singleplex	*cremoris*	1756	4.1	
P_lac_	Duplex	*lactis*	1480	6.2	2.6
	Singleplex	*lactis*	1442	7.1	
P_*tot*_	Duplex with P_lac_	*lactis*	1494	6.1	4.6
	Singleplex	*lactis*	1426	11	
	Duplex with P_cre_	*cremoris*	1748	5.1	1
	Singleplex	*cremoris*	1730	2.3	

*For duplex tests, P_*cre*_ and P_*lac*_ probes were in the presence of P_*tot*_ probe. Concentrations correspond to values in the original DNA sample tested. The range of DNA concentrations was selected to ensure the theoretical number of copies per microliter included in the non-saturating range of target recommended for ddPCR experiments. The coefficient of variation, in%, was calculated from five technical replicates following this formula: (standard deviation/average) × 100. Deviation between singleplex and duplex values, expressed as the% of the duplex concentration, is the ratio of the difference between singleplex and duplex average concentrations on the duplex average value. The *cremoris* and *lactis* species targets tested were, respectively, HER1205 and EIP33F strains.*

#### Limits of Detection (LoD) and Quantification (LoQ)

Limits of detection and quantification were determined by testing five DNA dilutions of a mixture containing the two species *lactis* (including the biovar diacetylactis) and *cremoris*. Each measurement was carried out in triplicate. For data analysis, the merge well function of QuantaSoft software was used to allow a more reliable, precise and accurate quantification based on “mega-wells” data, thus taking both intra- and inter-well variability into account.

As no significant differences were found between singleplex and duplex assays, P_lac_ and P_cre_ probes were used in duplex with P_*tot*_ probe. LoD corresponds to the lowest value of copies per microliter detected. It was defined as at least five positive droplets detected per well since the maximum number of positive droplets detected in negative controls was 4 (Table 4). For all probes, LoD was reached at dilution 3 with 11.6, 18.2, 9.5, 17.2 and 3.6 copies/μl, respectively, for P_lac_, P_*tot*_ (duplex with P_lac_), P_cre_, P_*tot*_ (duplex with P_cre_) and P_cit_ probes (Table 4). LoQ corresponds to the lowest target concentration quantified when specific positive droplets detected allow an accurate and reliable Poisson-estimated value with a deviation of the 95% confidence interval values from the estimated value <25% (Table 4). The 95% confidence interval is calculated from three technical replicates of merged wells. For P_lac_, P_cre_ and P_cit_ probes, LoQ is reached at dilution 2, respectively, with 98, 74 and 26.1 copies/μl present in the tested dilution. Indeed, at next dilutions, numbers 3, 4 and 5, the deviation of the highest and lowest values of the 95% CI from the estimated value was >25% (Table 4). For P_*tot*_, the deviation of the highest value of the 95% CI from the Poisson estimated concentration was > 25% (26% for P_*tot*_ in duplex with P_lac_ and with P_cre_) but the deviation of the lowest value of the 95% CI from the estimated concentration was <25% (22% for P_*tot*_ in duplex with P_lac_ and with P_cre_). As the strength of duplex assays is obtaining a reliable and accurate ratio value of *lactis* or *cremoris* targets for the entire *Lactococcus* population, LoQ values of 176 and 166 copies/μl for P_*tot*_ in duplex with P_lac_ and P_cre_, respectively, were retained, thus ensuring a quantification variability strictly below 25%.

**TABLE 4:** Determination of LoD and LoQ values for the four probes.

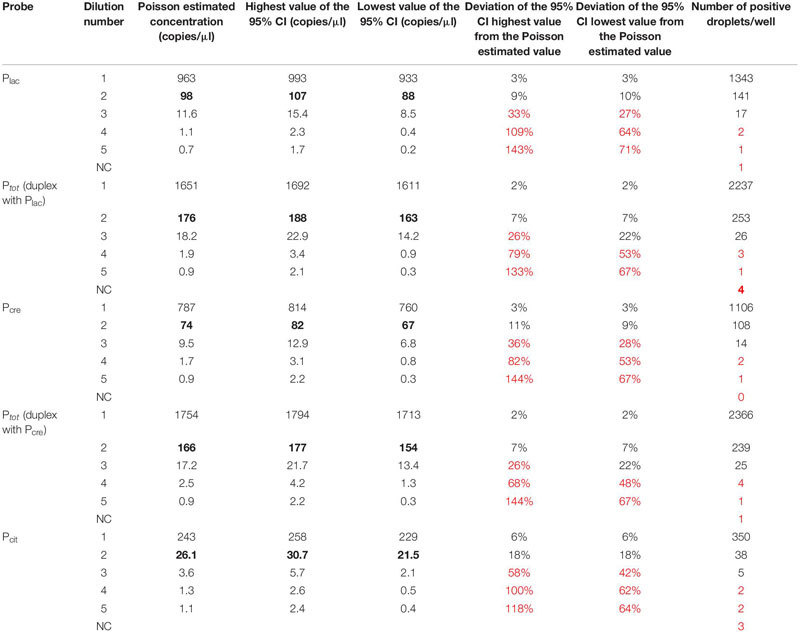

*Dilution numbers correspond to the five dilutions tested with a factor of 10 between each. NC stands for Negative Control tested for each duplex (P_*lac*_/P_*tot*_ and P_*cre*_/P_*tot*_) or singleplex (P_*cit*_) assay. The number of positive droplets per well is given for each NC and was reduced, for each sample, to a single well value, based on the merged values of three wells: the number of positive droplets from each “mega-well” was divided by the ratio of the total number of accepted droplets of the “mega-well” to the total number of accepted droplets of the “more positive” NC. The positive threshold is above 4 positive droplets per well, with values < 4 in red. LoQ values are in bold, with their corresponding 95% confidence intervals. Percentage deviation of the highest and lowest values of the 95% CI from the Poisson estimated value > 25% are in red.*

#### Test Validation on Reconstituted Starters

**FIGURE 2:**
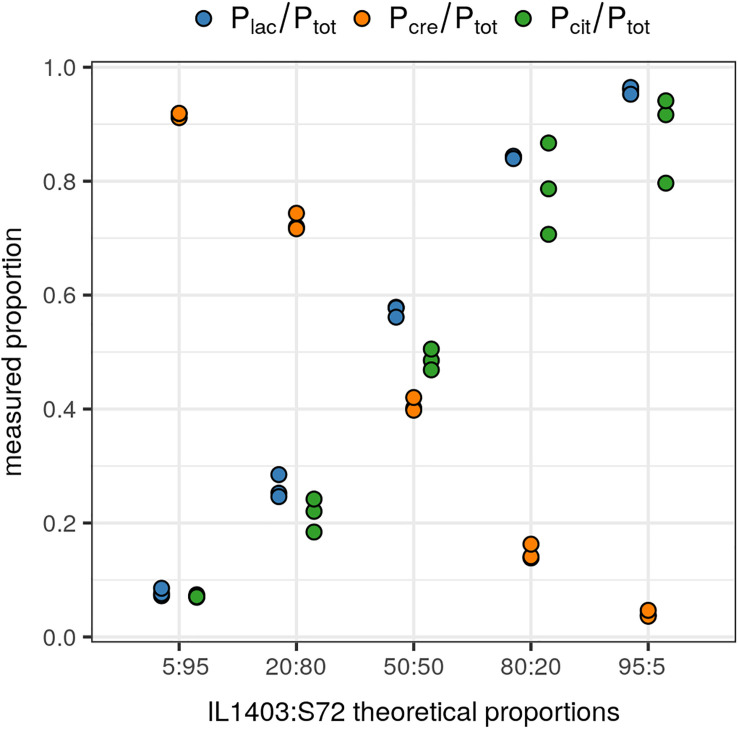
Relative quantification of the *lactis* and *cremoris* species and the biovar diacetylactis in reconstituted starters. Quantified proportions of *lactis* species and the biovar diacetylactis (IL1403 strain), and *cremoris* species (S72 strain), on the total *Lactococcus* population, in reconstituted starters with theoretical ratios of IL1403 to S72 of 5:95, 20:80, 50:50, 80:20, and 95:5. Data points represent ratios from three independently repeated experiments. P_lac_: *lactis* species-specific probe; P_cre_: *cremoris* species-specific probe; P_cit_: biovar diacetylactis-specific probe; P_*tot*_: *L. lactis* group-specific probe. Each P_lac_/P_*tot*_ and P_cre_/P_*tot*_ ratio was calculated from a duplex single well assay and P_cit_/P_*tot*_ ratio was obtained from the values of two independent wells.

The accuracy and reliability of the FAM/HEX ratios were validated on three independently repeated experiments using reconstituted starters with known proportions of each species and the biovar. IL1403 (species *lactis* biovar diacetylactis) and S72 (species *cremoris*) strains were mixed at ratios of IL1403 to S72 of 5:95, 20:80, 50:50, 80:20, and 95:5. For each mixture, the attempted proportions of IL1403 and S72 strains were confirmed with both duplex assays P_lac_/P_*tot*_ and P_cre_/P_*tot*_ (Figure 2). Moreover, IL1403 strain proportions were also confirmed with the P_cit_ probe. These results highlight the accurate and reliable detection and relative quantification of the two species and the biovar with this test.

### Monitoring *Lactococcus* Populations During the Milk Fermentation Process: Effect of Temperature and pH on the Balance of the Two Species and the Biovar

**FIGURE 3:**
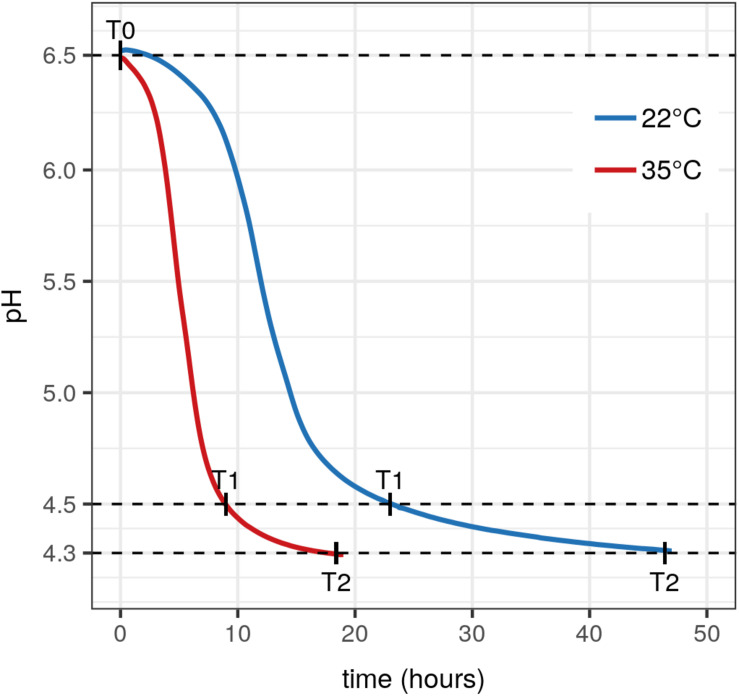
Acidification kinetics of MM100 starter during milk fermentation. Acidification curves of MM100 during milk fermentation at 22°C (blue curve) and 35°C (red curve). Each curve is the average of two pH kinetics from two independent cultures. T0, start of the fermentation after inoculation, T1 and T2, times when pH reached 4.5 and then 4.3.

**FIGURE 4:**
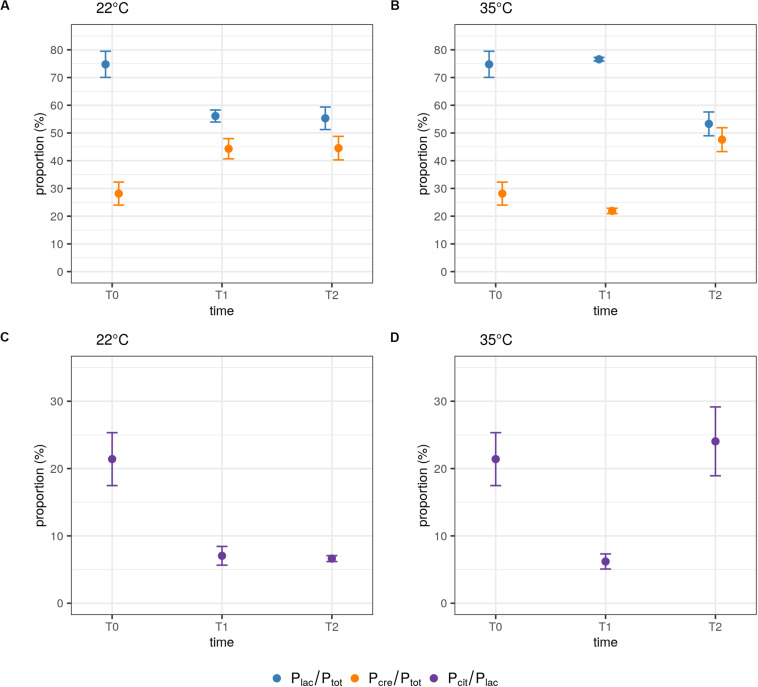
Influence of temperature and pH on the populations of the *lactis* and *cremoris* species and the biovar diacetylactis during the milk fermentation process. Quantified ratios P_lac_/P_*tot*_ and P_cre_/P_*tot*_ at 22°C **(A)** and 35°C **(B)**, and P_cit_/P_lac_ at 22°C **(C)** and 35°C **(D)** during growth. Each P_lac_/P_*tot*_ and P_cre_/P_*tot*_ ratio was calculated from a duplex single well assay and P_cit_/P_*tot*_ ratio was obtained from values from two independent wells. Samples were taken at the start of the fermentation (T0) and when pH reached 4.5 (T1) and then 4.3 (T2). Data points represent the mean and error bars represent the standard deviation of quantified ratios calculated from *n* independently repeated experiments, at T0, *n* = 6; at T1, *n* = 8 and *n* = 9 at 22°C and 35°C, respectively; and at T2, *n* = 6 at each temperature.

During the milk fermentation process, MM100 starter was described for *Lactococcus* populations considering temperature and pH conditions. The proportions of the two species *lactis* and *cremoris* were measured among the total *Lactococcus* population, and the biovar diacetylactis was analyzed regarding *lactis* species population. Table 5 lists descriptive statistics for the populations quantified. Acidification kinetics were also measured, and the pH curves revealed different activity depending on the temperature (Figure 3), with maximum acidification rates of −0.282 and −0.48 UpH/h at 22°C and 35°C, respectively. T0 refers to the initial state before growth, and T1 and T2 correspond to fermentation times when pH reached 4.5 and 4.3, respectively. At 22°C, T1 was reached after 22 h of fermentation and T2 after 47 h, and at 35°C, T1 was reached after 9 h and T2 after 19 h (Figure 3). We considered the cells remained under acid stress between T1 and T2. Figure 4 shows changes in the populations of *lactis* and *cremoris* species and biovar diacetylactis during the milk fermentation process under different temperature and pH conditions. The results of the ANOVA and of the *post hoc* test for the contrast of interest are listed in Supplementary Table S3. At T0, the mesophilic starter was dominated by the *lactis* species strains (75%). Among this population, 21% belonged to biovar diacetylactis. Strains of *cremoris* species represented 28% of the total *Lactococcus* population (Figures 4A–D and Table 5).

**TABLE 5:** Descriptive statistics for the quantified populations of the *lactis* and *cremoris* species and the biovar diacetylactis during the milk fermentation at two temperatures.

		**P_lac_/P_*tot*_**			**P_cre_/P_*tot*_**			**P_cit_/P_lac_**		
**Condition**	**Time**	***n***	***Mean***	***SD***	***n***	***Mean***	***SD***	***n***	***Mean***	***SD***
Initial										
	T0	6	0.75	0.047	6	0.28	0.042	6	0.21	0.039
22°C										
	T1	8	0.56	0.022	8	0.44	0.036	8	0.07	0.014
	T2	6	0.55	0.041	6	0.45	0.042	6	0.07	0.004
35°C										
	T1	9	0.77	0.007	9	0.22	0.01	9	0.06	0.011
	T2	6	0.53	0.043	6	0.48	0.043	6	0.24	0.051

*P_*lac*_/P_*tot*_, P_*cre*_/P_*tot*_ and P_*cit*_/P_*lac*_ ratios were quantified at the start of the fermentation (T0) and when pH reached 4.5 (T1) and then 4.3 (T2) during growth at two different temperatures, 22°C and 35°C. Each P_*lac*_/_*Ptot*_ and P_*cre*_/P_*tot*_ ratio was calculated from a single well of a duplex assay and the P_*cit*_/P_*lac*_ ratio was obtained from values of two independent wells. The number of independently repeated experiments (*n*), the mean and standard deviation (*SD*) of the ratios are reported.*

At 35°C, there was no statistically significant difference in the proportions of either *lactis* or *cremoris* species between T0 and T1 (*p* = 1 and *p* = 0.083, respectively). This confirms that the P_lac_/P_*tot*_ and P_cre_/P_*tot*_ ratios are conserved under recommended conditions of use. At 22°C, the balance of the two species between T0 and T1 was significantly modified (*p* < 0.001) with P_lac_/P_*tot*_ and P_cre_/P_*tot*_ ratios of 56% and 44%, respectively (Figures 4A,B and Table 5). At this temperature, the growth of *cremoris* species seems to be promoted whereas there was a decrease in populations of *lactis* species, probably leading to modified organoleptic features in the fermented product.

At 35°C, after a period of acid stress (T2), the population balance was significantly modified compared to in T1, with respectively, 53% and 48% of *lactis* and *cremoris* species (*p* < 0.001). In this starter, *cremoris* species strains were seen to be more resistant to the acid stress than *lactis* species strains. In contrast, at 22°C, no statistically significant effect of pH on the population balance was found, the proportions under acid stress conditions remained approximately the same with P_lac_/P_*tot*_ = 55% and P_cre_/P_*tot*_ = 45% (*p* = 1). A two-way ANOVA on P_lac_/P_*tot*_ and P_cre_/P_*tot*_ ratios revealed a significant interaction effect between pH and temperature [*F*(1,25) = 105.14, *p* < 0.001 and *F*(1,25) = 100.54, *p* < 0.001, respectively]. At T1, a significant difference between the ratios was found between 22°C and 35°C (*p* < 0.001, for both ratios); however, at T2, the temperature had no significant effect on P_lac_/P_*tot*_ and P_cre_/P_*tot*_ ratios (*p* = 0.633 and *p* = 0.413, respectively; Figures 4A,B).

The aromatic signature is due to the biovar diacetylactis strains, which represented 21% of the *lactis* species strains and hence 15% of the total population. At T1, the percentage of these strains dropped significantly to 7% and 6% of the *lactis* species population at 22°C and 35°C, respectively (*p* = 0.006, Figures 4C,D). In contrast, under acid stress conditions (T2), the percentage of biovar diacetylactis strains depended on temperature. At 35°C, this proportion increased to 24% (*p* = 0.012) suggesting a greater capacity for diacetyl production at this step of the process, while at 22°C, pH had no significant effect on P_cit_/P_lac_ ratio (*p* = 1), and the percentage remained at 7% (Figures 4C,D).

## Discussion

*L. lactis* and *L. cremoris* are widely used in starters for fermented dairy foods. As the two species have distinct genomic and phenotypic features, their respective proportions must be finely tuned throughout the design of the starters. Interactions between the two species probably occur during dairy fermentation, and the technological process used could change the balance between populations, thereby affecting the properties of the final product (Mills et al., 2010; Kelleher et al., 2017). Moreover, among *lactis* species, the biovar diacetylactis determines the specific aroma through the production of diacetyl. It is thus of primary importance to monitor each population’s dynamics. Different species- and biovar-specific PCRs have been proposed (Beimfohr et al., 1997; Corroler et al., 1999). qPCR assays using the *tuf* gene as target have been developed to quantify *L. lactis* group in cheese samples (Achilleos and Berthier, 2013). However, this method cannot selectively distinguish between *lactis* and *cremoris* species and the biovar. PCR-RFLP of the glutamate decarboxylase gene (*gadB*) has been proposed to distinguish the two species (Nomura et al., 2002), but this does not allow quantification and requires the strains to be isolated. With this aim in view, we identified three specific SNPs in the *gadB* gene and used them to design two species-specific probes. A third probe, *lactis* group-specific, in the same amplified fragment, enabled us to calculate the ratio of each species among the total population of *L. lactis* group in a duplex reaction. Both sets of primers and probes were tested on a panel of *L. lactis* and *L. cremoris* strains as well as on the species usually found in milk and dairy products. The target DNA was easily differentiated from the non-target DNA. As the populations were quantified, LoQ and LoD were defined for all the probes by testing several dilutions of a fermented milk sample. Depending on the probe, LoD values ranged from 3.6 × 10^3^ to 1.8 × 10^4^ copies/ml and LoQ values were 10 times higher. Considering the milk matrix, our results agree with the dynamic ranges of target determination encountered in dairy matrices like cheese using quantitative PCR (Zago et al., 2009).

Biovar diacetylactis is usually used by dairy manufacturers due to its capacity to produce diacetyl and acetoin, two aroma compounds associated with creamy and buttery flavors. The *citP* gene is highly valued as a molecular marker of this biovar. But the plasmid encoding CitP is sometimes missing while the chromosomal cluster *citDEFXG* is highly conserved among the strains that produce diacetyl (Passerini et al., 2013; Torres Manno et al., 2018). For that reason, the *citD* chromosomal gene encoding the citrate lyase was chosen as target to highlight the diacetylactis biovar. As few sequences are currently available in the databases, it will be necessary to update primers and probe with data accumulated in the coming years.

The specific objective of this study was to demonstrate that the *gadB* and *citD* targets described above can be used in digital droplet PCR assays to determine the proportions of the two species and the biovar in a starter or a dairy product. In contrast to qPCR, no standard curve with an external DNA calibrator is required and ddPCR shows little susceptibility to the inhibitors usually present in food matrices like dairy products (Morisset et al., 2013). With this approach, we analyzed species variations in a mesophilic starter called MM100 entirely composed of *Lactococcus* strains. First, we confirmed the microbial composition of the starter with 75% and 28% of *lactis* and *cremoris* species, respectively. Biovar diacetylactis was present at a ratio of 15% of the whole starter, a conventional ratio for such starters (Carr et al., 2002). Next, we analyzed variations in these ratios during the fermentation process as a function of the temperature and the pH. At the temperature specified by the supplier (35°C), pH 4.5 was reached after only 9 h and the proportions of the two species did not change substantially. Usually at this temperature, growth of strains of the *cremoris* species with a Cremoris phenotype (the “true Cremoris”) is not promoted (Cavanagh et al., 2015b). Since this species is maintained, we hypothesize that these strains have a Lactis phenotype. As reported in the literature, the large metabolic capabilities of the *lactis* species, particularly in carbohydrate metabolism, may promote faster growth rate than the *cremoris* species (Kelleher et al., 2017). However, as lactose is the main carbohydrate in milk, this dairy environment is very well adapted to the *cremoris* species strains which have the necessary ability to break down this sugar (Wels et al., 2019). Moreover, several studies have shown that strains of the *lactis* species initially produce acid slowly (Herreros et al., 2003; Fusieger et al., 2020) and that, conversely, strains of the *cremoris* species usually produce better acidification than *lactis* species, both at the beginning and at the end of the fermentation (Nomura et al., 2006; Fernández et al., 2011). Hence, we hypothesize that strains of the *cremoris* species significantly reduce the pH during fermentation.

At 22°C, the ratios were modified, with respectively, 56% and 44% of *lactis* and *cremoris* species. *cremoris* species growth seems promoted at this temperature, counter to *lactis* species whose proportion decreased. This change probably has an impact on the characteristics of the final product, resulting in different organoleptic characteristics from those obtained at 35°C. Indeed, starters with a majority of strains of the *cremoris* species can cause less bitterness in some dairy products like Cheddar cheese (Lee et al., 1996) compared to dairy products containing more *lactis* strains. This could be explained by different proteolytic systems in the two species. Comparative genome analysis identified an additional copy of *oppACBFD* operon encoding an oligopeptide transporter in most strains of the *cremoris* species, whereas only one operon has been described in most strains of the *lactis* species, and its functionality is unclear due to the presence of pseudogenes (Wels et al., 2019). Multiple genes for oligopeptides uptake should ensure more efficient degradation of peptides derived from milk.

Moreover, several strains of the *cremoris* species usually used in dairy starters display higher aminotransferase activity than their *lactis* counterparts (Kelleher et al., 2017). This is of particular importance in the fermented food industry because aminotransferases help developing flavor.

During acid stress, the proportions of the two species changed compared to their original proportions. Nevertheless, whatever the temperature, a balance was reached, and both species were present at the end of the process. This result is in agreement with the results of previous studies describing the persistence of the two species during cheese aging (Ruggirello et al., 2016). Moreover, *L. lactis* species is known to adapt its tolerance to acid stress conditions, especially by inducing responses during the exponential growth phase, allowing its survival under very low pH challenges (Frees et al., 2003). It is also worth noting that the proportion of biovar diacetylactis strains increases during the acid stress at 35°C. If this population is metabolically active at low pH, the citrate transport and its sequential conversion into pyruvate is probably induced (Martín et al., 2004). Excess pyruvate is rerouted toward the production of diacetyl/acetoin that adds flavor to the final product (García-Quintáns et al., 2008). This pathway confers a competitive advantage in condition of pyruvate accumulation (Zuljan et al., 2014). These aroma compounds are neutral metabolites and their synthesis contributes to pH homeostasis, which could explain the increase in the ratio of diacetylactis to *lactis* species at low pH. However, this increase does not occur at 22°C, suggesting that the lower temperature may adversely affect the activity of the enzymes involved in this pathway. Nevertheless, it should be borne in mind that differentiating between DNA of live and dead cells is a main issue when detecting by culture-independent based molecular methods. The majority of disrupted dead cells was discarded in the supernatant during the centrifugation preceding the DNA extraction. To ensure the detection of intact cells only, the use of ethidium monoazide (EMA) or propidium monoazide (PMA) based on membrane integrity could be a possible optimization of the test. Moreover, dairy process could induce VNC state as already described for *L. lactis* group (Ruggirello et al., 2018). A RNA-based analysis such as RT-ddPCR will discard metabolically inactive populations.

## Conclusion

We developed a classical ddPCR assay with fluorescent probes illustrating a proof of concept for the discrimination of populations of interest in dairy ecosystems. The primary objective of our work was to target the *lactis* and *cremoris* species and the biovar diacetylactis that would be of considerable industrial interest. We demonstrated the applicability of the ddPCR to quantify these populations in starters and to monitor their variations during the fermentation step of a technological process like cheese making. This technology showed high tolerance to inhibitors arising from food matrices and one duplex assay allowed us directly to provide the ratio of each population.

Finally, this methodology could easily be adapted to identify different cellular states and specific sub-populations in complex ecosystems like raw milks or cheeses, thereby completing 16S metagenomics studies.

## Data Availability Statement

All datasets presented in this study are included in the article/Supplementary Material.

## Author Contributions

M-LD-M, HT, and MC-B conceived the study. M-AC conceived the ddPCR design. MAb, MAu, and M-AC performed the experiments. IG performed the statistical analysis. M-LD-M, M-AC, HT, and MC-B analyzed the data and wrote the manuscript. All authors read, corrected, and approved the final manuscript.

## Conflict of Interest

FG was employed by CNIEL.

The remaining authors declare that the research was conducted in the absence of any commercial or financial relationships that could be construed as a potential conflict of interest.

## Footnotes

1 http://www.ncbi.nlm.nih.gov

2 https://eu.idtdna.com/pages

3 https://blast.ncbi.nlm.nih.gov/Blast.cgi
