# Supplementary material for: Precise Populations’ Description in Dairy Ecosystems Using Digital Droplet PCR: The Case of L. lactis Group in Starters
Source: Front Microbiol. 2020 Aug 6;11:1906. doi: 10.3389/fmicb.2020.01906 (PMC7423877; doi:10.3389/fmicb.2020.01906)
Supplement: Supplementary file 1 [file Data_Sheet_1.PDF]

SUPPLEMENTARY MATERIAL

Table S1: ***Lactococcus lactis*** **and** ***cremoris*** **strains used for the sequences alignment of the** ***gadB*** **gene to design primers and probes for ddPCR assays.**

**Strain**	**Accession number or WGS**	***Species***
NCDO2118	CP009054.1	*lactis*
NCDO2727	Unpublished results	*lactis*
KF147	CP001834	*lactis*
KLDS4.0325	CP006766	*lactis*
FM03	CP020604	*lactis*
UC77	CP015906	*lactis*
UL8	CP015908	*lactis*
UC063	CP015905	*lactis*
229	CP015896	*lactis*
IL1403	CP033607	*lactis*
C10	CP015898	*lactis*
CV56	CP002365	*lactis*
275	CP015897	*lactis*
184	CP015895	*lactis*
A12	LT599049	*lactis*
S0	CP010050	*lactis*
UC11	CP015904	*lactis*
UC08	CP015903	*lactis*
IO-1	NC_020450	*lactis*
UC06	CP015902	*lactis*
AI06	CP009472	*lactis*
NZ9000	CP002094	*cremoris*
MG1363	NC_009004	*cremoris*
EIP31B	Unpublished results	*cremoris*
EIP36H	Unpublished results	*cremoris*
NCDO763	LITG01	*cremoris*
N41	LITA01	*cremoris*
MET520	Unpublished results	*cremoris*
MET521	Unpublished results	*cremoris*
MET523	Unpublished results	*cremoris*
GE214	AZSI01	*cremoris*
DPC6856	LAVW01	*cremoris*
KW2	CP004884	*cremoris*
A17	JQIC01	*cremoris*
V4	LIYG01	*cremoris*
Mast36	JZUI01	*cremoris*
DPC6860	LAVX01	*cremoris*
MET516	Unpublished results	*cremoris*
MET233	Unpublished results	*cremoris*

UC509.9	CP003157	*cremoris*
158	CP015894	*cremoris*
UC109	CP015907	*cremoris*
SK11	CP000425	*cremoris*
JM3	CP015901	*cremoris*
A76	CP003132	*cremoris*
JM2	CP015900	*cremoris*
JM1	CP015899	*cremoris*
JM4	CP015909	*cremoris*
MET539	Unpublished results	*cremoris*
MET555	Unpublished results	*cremoris*
MET542	Unpublished results	*cremoris*
MET541	Unpublished results	*cremoris*
HP	LIYE01	*cremoris*
LMG6897	LISZ01	*cremoris*
AM2	LITE01	*cremoris*
FG2	LITD01	*cremoris*
WG2	LXWJ01	*cremoris*

Table S2: *Lactococcus lactis* biovar diacetylactis strains used for the sequences alignment of the *citD* gene to design primers and probe for ddPCR assays.

Strain	Accession number or WGS
IL1403	NC002662.1
FM03	CP020604.1
UC77	CP015906.1
229	CP015896.1
184	WJUU01
CRL264	LKPE01
LMG19460	MUBH01000001.1
DRA4	LIWD01000232.1
TIFN2	ATBF01000049.1
TIFN4	ATBD01000019.1
LD61	AXZK01000045.1

Table S3: ANOVA results examining the influence of temperature and pH on the populations of the *lactis* and *cremoris* species and the biovar diacetylactis during milk fermentation.

		one-way ANOVA		post-hoc test	
**Condition**	**ratio**	**F**	**p value**	**T1 vs. T0**	**T2 vs. T1**
**22°C**					
	P_{lac}/P_{tot}	F(2,17)=57.02	<0.001	<0.001	1.000

	P_{cre}/P_{tot}	F(2,17)=35.10	<0.001	<0.001	1.000
	P_{cit}/P_{lac}∗	F(2,8.59)=38.96	<0.001	0.006	1.000
**35°C**					
	P_{lac}/P_{tot}	F(2,18)=95.56	<0.001	1.000	<0.001
	P_{cre}/P_{tot}	F(2,18)=118.19	<0.001	0.083	<0.001
	P_{cit}/P_{lac}∗	F(2,7.22)=68.64	<0.001	0.006	0.012

		**two-way ANOVA**		**post-hoc test**	
				**35°C vs. 22°C**	
**Ratio**	**term**	**F**	**p value**	**T1**	**T2**
**P_{lac}/P_{tot}**					
	Temperature	F(1,25)=113.46	<0.001		
	pH	F(1,25)=126.69	<0.001		
	Interaction	F(1,25)=105.14	<0.001	<0.001	0.633
**P_{cre}/P_{tot}**					
	Temperature	F(1,25)= 95.67	<0.001		
	pH	F(1,25)=109.27	<0.001		
	Interaction	F(1,25)=100.54	<0.001	<0.001	0.413

∗ Welch's ANOVA and Games–Howell post–hoc test

P_{lac}/P_{tot}, P_{cre}/P_{tot} and P_{cit}/P_{lac} ratios were quantified at the start of the fermentation (T0) and when pH reached 4.5 (T1) and then 4.3 (T2), at two different temperatures, 22 °C and 35 °C. Data exhibiting unequal variance across times T0, T1 and T2 were analyzed using a Welch's ANOVA combined with a post-hoc Games-Howell test. P-values from the contrast of interest are reported.
